# Mechanism-guided fine-tuned microbiome potentiates anti-tumor immunity in HCC

**DOI:** 10.3389/fimmu.2023.1333864

**Published:** 2023-12-19

**Authors:** Tao Liu, Ya Guo, Yanxia Liao, Jinping Liu

**Affiliations:** State Key Laboratory of Oncology in South China, Guangdong Provincial Clinical Research Center for Cancer, Sun Yat-sen University Cancer Center, Guangzhou, China

**Keywords:** HCC TME, immunotherapy, microbiome, bacteria, fungi, viruses, cell-type niche, microbe-host interplay

## Abstract

Microbiome, including bacteria, fungi, and viruses, plays a crucial role in shaping distal and proximal anti-tumor immunity. Mounting evidence showed that commensal microbiome critically modulates immunophenotyping of hepatocellular carcinoma (HCC), a leading cause of cancer-related death. However, their role in anti-tumor surveillance of HCC is still poorly understood. Herein, we spotlighted growing interests in how the microbiome influences the progression and immunotherapeutic responses of HCC via changing local tumor microenvironment (TME) upon translocating to the sites of HCC through different “cell-type niches”. Moreover, we summarized not only the associations but also the deep insight into the mechanisms of how the extrinsic microbiomes interplay with hosts to shape immune surveillance and regulate TME and immunotherapeutic responses. Collectively, we provided a rationale for a mechanism-guided fine-tuned microbiome to be neoadjuvant immunotherapy in the near future.

## 1 Introduction

Liver cancer is a substantial global health issue, ranking fourth in cancer-related deaths and sixth in incident cases worldwide (1). Primary liver cancer, specifically hepatocellular carcinoma (HCC), is the most common type, accounting for numerous cancer-related deaths globally. The 5-year relative survival rate for HCC is only 18% worldwide, which is even lower in China (12.5%) (2, 3). HCC often develops from chronic liver diseases, such as hepatitis B and C virus infections, alcohol abuse, and fatty liver disease (2, 4). These factors can lead to hepatitis, which may irreversibly progress to HCC (5). In recent years, there have been advancements in the early diagnosis of HCC, which is crucial in preventing mortality (3). Currently, most liver cancer patients are diagnosed at an advanced stage, resulting in a high mortality rate. Traditionally, ultrasonographic (US) surveillance and serological assessment of alpha-fetoprotein (AFP) have been used for early-stage diagnosis of HCC (6). However, the specificity and sensitivity of US/AFP are inadequate for detecting HCC in its early stages. Nevertheless, a breakthrough study by Liu et al. utilized the VirScan method to identify a novel virus exposure signature that serves as an effective cancer biomarker for early-onset HCC (7). Notably, this new biomarker outperformed AFP significantly. Therefore, these recent technological advancements offer promising prospects for early detection of HCC, which is essential for improving patient outcomes.

As we all know, human skin and mucosal tissue are colonized by numerous microbiome, which can be functionally divided into three categories: symbiotic, probiotic, and pathogenic microbiome. Approximately 4 × 10^13^ microbial cells spanning ~3 × 10^3^ species inhabit the human body. Most (97%) of them are bacteria in the colon, and the fungi only represent 0.1%–1% (8). This microbiome maintains a dynamic balance with human tissues and has various functions, including metabolism (9), biological barrier maintenance (10), and immune regulation (11). Furthermore, commensal microbiome can influence the development of several diseases, such as obesity (12), diabetes (13), hypertension (14), inflammatory bowel disease (15), liver disease (16), cancer (8, 17), neurological disease (18), and autoimmune disease (19). Some studies suggest that microbiome can affect the host’s biological clock and are considered vital immune organs (20, 21).

Recent pan-cancer studies have demonstrated a close association between bacteria, fungi, and cancer (22–25). Researchers analyzed genomic and transcriptomic data from The Cancer Genome Atlas of 33 cancer types and identified unique microbial signatures in tissues and blood samples (22). Another study employed bacterial 16s rRNA gene PCR technology and visualization techniques to characterize the bacterial presence in tumor tissues. They found distinct microbiome compositions in each cancer type, and the intratumoral bacteria are mostly intracellular and are present in both cancer and immune cells (23). Similarly, the study analyzed the cancer mycobiome across 35 cancer types and observed differences in fungal composition. The presence of fungi within tumors were confirmed by histological staining, often in proximity to cancer cells and macrophages (24). Specifically, gastrointestinal tumors were associated with *Candida* species, *Saccharomyces* cerevisiae, and *Cyberlindnera jadinii*, while lung and breast tumors showed abundant *Blastomyces* and *Malassezia* species, respectively. Gastric cancer exhibited increased expression of pro-inflammatory immune pathways linked to *Candida* presence (25). Tumor-associated microbiome can be found in various locations, including blood vessels, intestines, and tumor tissues. Collectively, the intracellular microbiomes were most likely found in the different cell type niches, such as localized within tumor cells and macrophages.

Moreover, commensal microbiome can affect the effectiveness of cancer immunotherapy and chemotherapy (26). For instance, in patients who received immune checkpoint inhibitor anti-programmed cell death 1 protein (PD-1) immunotherapy, there were significant differences in the intestinal microbiome of responders and non-responders, mainly in diversity and composition (27). At the same time, fecal bacterial transplantation (FMT) combining immune checkpoint inhibitors has been clinically used in the treatment of refractory melanoma patients. Further studies have found that FMT changes the tumor microenvironment and enhances immune cell infiltration in melanoma patients. In short, FMT overcomes resistance to anti-PD-1 immunotherapy in advanced melanoma patients (28, 29). Recent, a mouse melanoma study identified a bacterium, *C. cateniformis*, that enhances anti-tumor immunity by downregulating PD-L2 expression and its binding partner, RGMb (30).

This review encompasses microbial alterations in liver cancer patients, the impact of commensal microbiome on liver cancer development, and their role in cancer immunotherapy. Based on these findings, potential recommendations are proposed for microbiome intervention in HCC immunotherapy.

## 2 Microbial characteristics of HCC

### 2.1 Bacteria reshape anti-tumor immunity in HCC

From the summary of existing studies (**Table 1**), it is evident that there is a close relationship between the commensal microbiome of the host and the occurrence and development of HCC. Most of the research has focused on fecal samples. Data on serum samples and liver tumor tissue samples also exist.

**Table 1:** Bacterial and fungal dysbiosis in HCC patients.

Category	Up	Down	Sample	Reference
**HCC/HC**	*Escherichia-Shigella* *Fusobacterium* *Megasphaera* *Veillonella* *Tyzzerella_4* *Prevotella_2* *Cronobacter*	*A Blautia* *Fusicatenibacter* *Anaerostipes* *Lachnospieaceae_ND3007_group* *CAG-56,Eggerthella* *Lachnospiraceae_FCS020_group* *Olsenella*	Feces	(31)
**HCC/HC**	*Staphylococcus* *Acinetobacter* *Trabulsiella* *Klebsiella*	*Pseudomonas*	Serum	(32)
**HCC/para-carcinoma-tissue**	*Enterobacteriaceae* *Fusobacterium* *Neisseria*	*Pseudomonas*	HCC tissue	(33)
**HCC/cirrhosis**	*Gemmiger* *Parabacteroides* *Paraprevotella* *Klebsiella* *Haemophilus*	*Ruminococcus* *Oscillibacter* *Faecalibacterium* *Clostridium IV* *Coprococcus* *Akkermansia*	Feces	(34)
**HCC/without-HCC**	*Erysipelotrichaceae* *Odoribacter* *Butyricimonas*	*Leuconostocaceae* *Fusobacterium* *Lachnospiraceae family genus Dorea*	Feces	(35)
**HCC/LC&HC**	*Enterobacter ludwigii*		Feces	(36)
**HCC stage I/HC**	*Actinomyces, Atopobium*, *Desulfococcus, Enterobacter, Paraprevotella, Planctomycetes, Prevotella, Veillonella*	*Acidaminococcus*, *Cetobacterium*, *Coprobacillus*, *Pyramidobacter*, *Turicibacter*	Feces	(37)
**HCC stage II/HC**	*Desulfococcus,Enterobacter*, *Lactococcus, Leptotrichia*, *Paraprevotella*, *Planctomycetes*, *Prevotella, Veillonella*	*Anaerotruncus* *Cetobacterium*		
**HCC stage III/HC**	*Actinomyces, Atopobium, Desulfococcus, Enterobacter, Haemophilus, Lactococcus, Leptotrichia, Neisseria, Oribacterium, Prevotella, Rothia, Selenomonas, Veillonella*	*Acidaminococcus*, *Anaerostipes, Anaerotruncus*, *Butyricimonas, Cetobacterium*, *Cloacibacillus, Coprobacillus*, *Holdemania, Methanobrevibacter, Odoribacter, Pyramidobacter*, *Turicibacter*		
**Early-stage HCC**	*Clostridiales, Firmicutes Streptococcus*	NA	Feces	(38)
**Intermediate-stage HCC**	*Ruminococcaceae, Pasteurellaceae, Tanticharoenia, and Vagococcus*	NA		
**Advanced-stage HCC**	*Bifidobacteriales, Actinobacteria, Barnesiella, Porphyromonadaceae, and Pseudomonadales*	NA		
**NBNC-HCC/HBV-HCC**	*Escherichia-Shigella* *Enterococcus*	*Faecalibacterium* *Ruminococcus* *Ruminoclostridium*	Feces	(39)
**HBV-HCC/HC**	*Bacteroides* *Lachnospiracea incertae sedis* *Clostridium XIVa*	NA	Feces	(40)
**HCC/cirrhosis&HC**	*Malassezia* spp. *Candida* spp.	NA	Feces	(41)

In a study comparing the gut microbiome of elderly HCC patients and healthy individuals using next-generation sequencing of fecal samples, both α diversity and β diversity were statistically different. At the genus level, several bacterial groups, including *A.Blautia*, *Fusicatenibacter*, *Anaerostipes*, *Lachnospiraceae_ND3007_group*, *CAG-56*, *Eggerthella*, *Lachnospiraceae_FCS020_group*, and *Olsenella* were significantly decreased in the HCC group compared to the control group. Conversely, the abundance of *E. coli-Shigella*, *Fusobacterium*, *Megalococcus*, *Veillonella*, *Tyzzerella_4*, *Prevotella_2*, and *Cronobacter* was significantly increased in the HCC group (31). Furthermore, the composition of bacteria in the serum of liver cancer patients differs from that of cirrhosis patients (32). *Pseudomonas* was found to be significantly reduced in HCC, whereas *Staphylococcus*, *Acinetobacter*, *Klebsiella*, and *Trabulsiella* were significantly enriched. The presence of intratumoral microbiome in HCC tumor tissues is of increasing concern (33), and significant differences in microbial composition have been observed between liver cancer tissues and para-cancerous tissues. Notably, the microbial diversity in liver tumor tissues is significantly higher than in para-cancerous tissues. The abundance of *Enterobacteriaceae*, *Fusobacteria*, *Neisseria*, and other microbiome in HCC tissues is considerably higher, while certain anti-tumor bacteria, such as *Pseudomonas* spp., is decreased.

Ninety percent of HCC cases arise from cirrhosis, during which liver cells undergo chronic cycles of necrosis and regeneration (42). Compared with patients with cirrhosis (34), those with early-stage liver cancer caused by cirrhosis showed increased intestinal microbiome diversity. Specifically, phylum *Actinobacteria* was significantly increased in early HCC. Correspondingly, 13 genera, including *Gemmiger*, *Parabacteroides*, and *Paraprevotella*, were enriched in early HCC compared to liver cirrhosis. On the other hand, phylum *Verrucomicrobia* was decreased in early HCC. At the genus level, 12 genera, including *Alistipes*, *Phascolarctobacterium*, and *Ruminococcus*, were significantly reduced, while six genera, including *Klebsiella* and *Haemophilus*, were increased in early HCC compared to controls. Notably, a reduction in butyrate-producing bacteria (*Ruminococcus, Oscillibacter, Faecalibacterium, Clostridium IV*, and *Coprococcus*) and an increase in lipopolysaccharide-producing bacteria (*Klebsiella* and *Haemophilus*) were observed. Butyrate is the primary energy source of the intestinal mucosa, playing a pivotal role in bacterial energy metabolism and intestinal health (43). Therefore, reducing butyrate-producing bacteria may contribute to intestinal mucosal destruction and the development of liver cancer (44). Increased LPS levels can activate the NF-κB pathway and produce pro-inflammatory cytokines, leading to liver inflammation and oxidative damage, promoting the development of HCC (45). Furthermore, research in Argentina found specific changes in the *Firmicutes* members and identified potential biomarkers of HCC, including *Odoribacter*, *Butyricomonas*, and *Lachnospiraceae family genus Dorea* (35).

In another study comparing primary liver cancer patients with cirrhosis patients and healthy individuals, the diversity of *Firmicutes* showed a downward trend from the healthy group to the liver cirrhosis group to the primary liver cancer group. *Enterobacter ludwigii* increased in the primary liver cancer group. Further analysis revealed correlations between clinical indicators and intestinal microbiome, such as a positive correlation between *Veillonella* and AFP in the primary liver cancer group and a negative correlation between *Subdolicapsulum* and AFP (36).

To administrate the best-fit treatment, it is indispensable for cancer stages. Usually, cancer staging is based on the size of the tumor, whether it has metastasized to lymph nodes, and whether it has metastasized to other organs. Different cancers have their unique staging system. Various staging systems used in HCC include the European systems [French staging system, Barcelona Clinic Liver Cancer (BCLC) staging system, and the Cancer of the Liver Italian Program (CLIP)] and Asian systems [Okuda staging system, Japan integrated Staging (JIS), Tokyo score and Chinese University Prognostic Index (CUPI)] (46). There were significant differences in the intestinal microbiome of patients at different stages of HCC (37). In 86 stool samples, 604 bacterial genera were identified. Compared with healthy controls, patients with stage I HCC showed enhancement of *Actinomyces, Atopobium, Desulfococcus, Enterobacter, Paraprevotella, Planctomycetes, Prevotella, Veillonella*, and many unidentified genera. Patients with stage II HCC exhibited enrichment of *Desulfococcus, Enterobacter, Lactococcus, Leptotrichia, Paraprevotella, Planctomycetes, Prevotella, Veillonella*, and many unknown genera. Patients with stage III HCC demonstrated multiplication of *Actinomyces, Atopobium, Desulfococcus, Enterobacter, Haemophilus, Lactococcus, Leptotrichia, Neisseria, Oribacterium, Prevotella, Rothia, Selenomonas, Veillonella*, and many unidentified genera. Moreover, *Desulfococcus, Enterobacter, Prevotella, and Veillonella* were increased in all stages of HCC. However, patients with stage I HCC showed a reduction in *Acidaminococcus, Cetobacterium, Coprobacillus, Pyramidobacter, Turicibacter*, and two unidentified genera; patients with stage II HCC exhibited a decrease in *Anaerotruncus, Cetobacterium*, and an unknown genus. Additionally, patients with stage III HCC showed a reduction in *Acidaminococcus, Anaerostipes, Anaerotruncus, Butyricimonas, Cetobacterium, Cloacibacillus, Coprobacillus, Holdemania, Methanobrevibacter, Odoribacter, Pyramidobacter, Turicibacter*, and four unidentified genera. *Cetobacterium* was reduced in all stages of primary HCC.

Another study tested 45 patients’ feces with early, intermediate, and late stages and found that the intestines of the early-stage liver cancer group had a higher abundance of *Clostridium, Firmicutes*, and *Streptococcus* (38). The mid-stage liver cancer group presented more intestinal *Ruminococcaceae, Pasteurellaceae, Tanticharoenia*, and *Vagococcus genera*. The advanced liver cancer group displayed more *Bifidobacteria, Actinobacteria, Barnella, Porphyromonas*, and *Pseudomonas*. In summary, during the development of liver cancer, there are significant differences in the composition of the intestinal flora of patients, in addition to changes in relevant clinical indicators.

HBV is one of the most critical factors causing HCC, accounting for approximately 50% of cases (47). Studies have compared the differences in intestinal microbiome between HBV-HCC and non-HBV non-HCV related HCC (NBNC-HCC) patients (39). Fecal samples from 90 patients were analyzed, revealing significant differences in the diversity and composition of the intestinal microbiome. Specifically, HBV-HCC patients had higher species richness, while NBNC-HCC patients had decreased *Firmicutes* and increased *Proteobacteria* at the phylum level. NBNC-HCC patients had fewer potential anti-inflammatory and pro-inflammatory bacteria, whereas HBV-HCC patients had more potentially anti-inflammatory bacteria. Overall, HBV-HCC and NBNC-HCC patients exhibited variations in the abundance of bacteria involved in different functions or biological pathways, suggesting that alterations in specific gut microbiome could have therapeutic benefits for both types of HCC. Another study compared gut microbiome differences between HBV-HCC patients and healthy individuals (40). The researchers found that HCC patients with high tumor burden had an enrichment of *Bacteroides*, *Lachnospiracea incertae sedis*, and *Clostridium XIVa*. Furthermore, a strong correlation was observed between the expression of these three genera of bacteria and the host tumor microenvironment genes. Based on further clinical characterization and database analysis, serum bile acids were identified as potential communication mediators between these bacteria and the host transcriptome. This study suggests that changes in the tumor immune microenvironment associated with intestinal microbiome, mediated by serum bile acids, might be crucial in tumor burden and adverse clinical outcomes in HBV-related HCC.

### 2.2 Fungi alters HCC immune microenvirnment

Numerous recent studies have investigated the contribution of fungi to the onset and progression of various tumors, such as those found in colorectal (48–55), pancreatic (56), skin (57), bladder (58), lung (59) cancers, and more. Meanwhile, Fungal colonization in cancer patients manifests in different tissue and cell type niches. One of the most recent pan-cancer study has detected fungi in 35 types of tumors, indicating that they may exist in tumor cells or immune cells, similar to intracellular bacteria (24). However, the mechanisms by which fungi enter these cells in tumor models and how they survive within them are still unknown.

As opportunistic pathogens, fungi pose a severe threat to people’s health, particularly those with compromised immune function, such as AIDS patients, tissue transplant recipients, or cancer patients (60), additionally, individuals with mutated antifungal immunity-related genes are prone to frequent mycoses (61). To evade the innate immune surveillance, fungi employ similar strategies in different diseases (62). Such as *Cryptococcus neoformans* has been reported to cross the blood-brain barrier by concealing itself within macrophages, leading to meningitis (63). And another example is *Candida albicans*, which can survive within macrophages by undergoing morphological transformation, forming hyphae, and compromising the integrity of the phagosomal membrane (64).

Stool samples from HCC and liver cirrhosis patients demonstrated decreased fungal diversity in the HCC group, with a higher abundance of *Candida* genus and *Candida albicans* (65). Similarly, patients with liver cancer showed reduced fungal diversity compared to the healthy group, with significantly higher abundances of *Candida* albicans and *Malassezia* in the feces of liver cancer patients. Studies on animals have verified that unusual growth of Candida albicans and Malassezia furfur in the gut can contribute to the onset of HCC (41).

### 2.3 Virome profiling reveals the pivotal virus-host interactions in HCC and other cancer types

Recent research that employs metagenomic sequencing and other techniques has given us a deeper understanding of the diversity of the human virome in various parts of the body, its connection to diseases, and how it forms during the early stages of life (66). This human virome is made up of bacteriophages, which are viruses that infect bacteria, as well as viruses that target other cellular microorganisms such as archaea, those that invade human cells, and those that are temporary components of food (67–73). Notably, the virome can elicit a broad range of immune responses (74).

**Figure 1:**
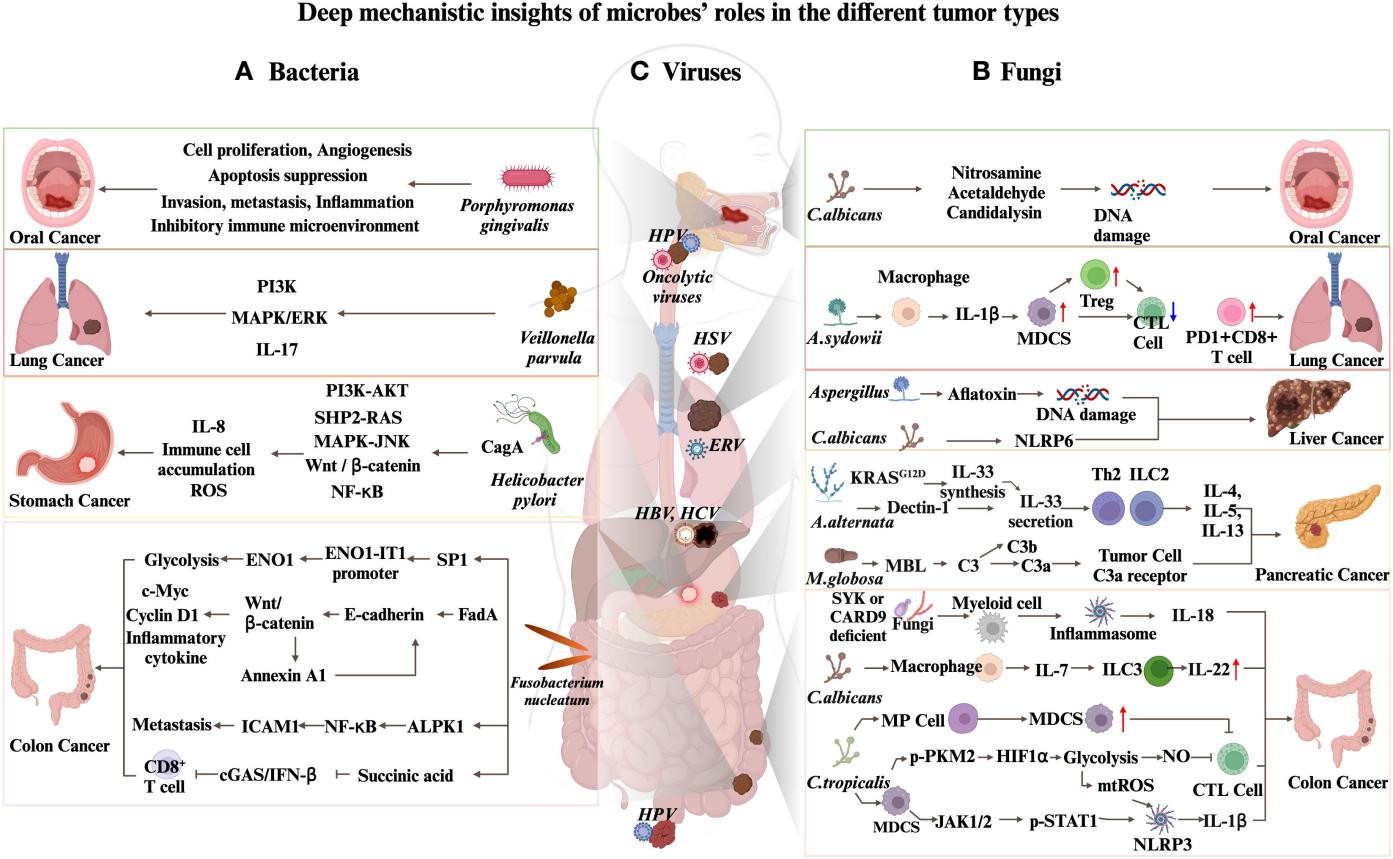
Deep mechanistic insights of microbes’ roles in different tumor types. **(A)** Mechanisms by which bacteria promote carcinogenesis. **(B)** Mechanisms by which fungi promote carcinogenesis. **(C)** Oncolytic viruses and ERVs contribute to cancers immunotherapy.

Various types of cancer have been linked to viral infections (**Figure 1C**). For example, human papillomavirus (HPV) is recognized as a requisite component in the formation of both pre-invasive and invasive lower genital tract cancers, with the most common being cervical cancer (75), and Malignant transformation of HPV can also cause head and neck squamous cell carcinoma (HNSCC) (76). Likewise, the Epstein-Barr virus (EBV) is viewed as a primary causal agent in the occurrence of nasopharyngeal cancer (77). HBV is identified as a partially double-stranded DNA virus, in contrast to HCV, a positive-sense single-stranded RNA virus. These viruses are known as the primary causal factors of HCC. Furthermore, other liver viruses, like hepatitis G virus, are also possible causative factors for HCC, even though their correlation has not been conclusively proven (78). Adeno-associated virus 2 has been reported to cause HCC (79), and next-generation sequencing technologies have uncovered associations of other viruses with HCC, suggesting that a range of viruses could be involved in its pathogenesis (80).

HCC virome study mapped 93,904 viral epitopes and identified the most variable viruses in tumor patients versus controls, utilizing large-scale screening of thousands of serological samples and machine learning approaches (7). It elucidated a viral exposure signature to define the early onset of HCC through the large-scale screening of thousands of human samples and applying pan-viral-epitope screening of human virome in HCC. This viral exposure signature (VES) was defined by comparing population controls and HCC patients, comprising unique epitopes from 61 viral strains (18 high-risk associated strains and 43 low-risk associated strains). Notably, 11 of the 18 high-risk associated strains were HCV, a well-established trigger of HCC, and were more prevalent in cases than in controls. The VES also included previously unknown viruses associated with HCC, with an enrichment of cytomegalovirus (CMV), hepatitis delta virus (HDV), and influenza strains H1N1 and H3N2. Emerging infectious diseases significantly burden national public health and may substantially influence tumor progression and therapeutic efficacy. Therefore, the virome profile can be the key biomarker for clinical characteristics and outcomes.

## 3 Microbiome contribute immunotherapy efficacy

In liver cancer and other cancers, the expression of corresponding ligands in tumor and stromal cells allows them to evade anti-tumor immune responses by exploiting the physiological mechanism of immune checkpoints, which include co-inhibitory molecules expressed by effector lymphocytes to prevent their overactivation (81). These co-inhibitory receptors, such as CTLA4, PD1, TIM3, and LAG3, are expressed by various cells, including T cells, natural killer cells, myeloid cells, and dendritic cells (82). CTLA4 is expressed by activated T cells and primarily by Treg cells. It blocks the activation of effector T cells and acts as an effector molecule for T cells (83). PD1 is expressed by activated T cells, natural killer (NK) cells, Treg cells, MDSCs, monocytes, and dendritic cells (DC). In contrast, its ligand PDL1 is expressed by stromal cells, tumor cells, and myeloid cells (such as DCs). PD1 inhibits effector function and leads to effector T-cell exhaustion or dysfunction. Immune checkpoint inhibitors (ICIs) are monoclonal antibodies that block the interaction of checkpoint proteins with their ligands, thereby preventing T-cell inactivation. Immune checkpoint inhibitors (e.g.,anti-PD1 or anti-CTLA4 antibodies) have revolutionized the treatment of many cancers (84, 85).

In the case of hepatocellular carcinoma (HCC) patients, anti-PD-1, anti-PD-L1, and anti-CTLA-4 monoclonal antibodies are commonly used ICIs in clinical practice. Combination strategies involving immunotherapy, such as anti-PD-1/PD-L1 mAbs plus anti-VEGF mAbs, TKIs, or anti-CTLA-4 mAbs, have also been widely used to overcome drug resistance and improve efficacy (86). However, there are challenges in HCC ICI treatment. The heterogeneity of HCC and its complex immunological microenvironment can influence variable responses to ICIs (87, 88). Biomarker discovery is crucial for identifying patients most likely to benefit from ICIs (89). Moreover, resistance to ICIs is a significant challenge in HCC treatment, and various resistance mechanisms have been identified (90). Overcoming the challenges requires a deep understanding of the mechanisms underlining how the pathophysiology of HCC interplay with the tumor microenvironment to fine-tune the responses to ICIs (91). Future research should focus on developing strategies to improve patient outcomes and increase the response rate to ICIs in HCC.

The gut microbiome also plays a significant role in modulating the efficacy of immunotherapy for cancer (92). Modulating the gut microbiome through techniques such as fecal transplantation, probiotics, consortia, and diet can enhance the clinical response rates to immunotherapy (93). Favorable modulation of the microbiome is associated with increased infiltration of CD8^+^ effector T cells into the tumor, leading to enhanced intratumoral activity of T-helper type 1 cells, dendritic cells, and a lower density of immunosuppressive cells (94). Disruptions in the gut microbiome can promote hepatocellular carcinoma development and influence the response to immunotherapy in the context of liver cancer (95). The gut microbiome may regulate the responses to immune checkpoint inhibitors in HCC patients (96). As a result, the gut microbiome has emerged as a prognostic biomarker and a potential therapeutic target to enhance the efficacy of immunotherapy.

### 3.1 Gut microbiome influencing the efficacy of HCC immunotherapy

Studies have focused on investigating the role of commensal microbiome in ICI treatment of HCC (97). Metagenomic sequencing has been used to detect the dynamic characteristics and specificity of the intestinal microbiome during anti-PD-1 immunotherapy in HCC. Stool samples from patients who responded to immunotherapy showed higher tax on richness and more gene counts than non-responders. The microbial composition of responders remained relatively stable at the phylum level and was enriched in *Myxobacteria* and *Ruminococcus* species, whereas Proteobacteria increased in non-responders (98).

Another study found that several specific taxa were significantly enriched in the clinical benefit response (CBR) group compared to the non-clinical benefit group (NCB). For example, patients with a higher abundance of *Lachnospiraceae bacteria - GAM79* and *Alistipes sp Marseille - P5997* had longer progression-free and overall survival times. On the other hand, patients with higher *Veillonellaceae* abundance had poorer progression-free and overall survival (99).

In a prospective analysis of fecal samples from unresectable HCC patients before immunotherapy, significant differences in fecal bacteria were observed between patients who responded to therapy and those who did not. Prevotella 9 was enriched in non-responders, while *Clostridium*, *Lachnospiraceae*, and *Veillonella* were dominant in responders. Furthermore, specific metabolites such as ursodeoxycholic acid and urscholic acid were significantly enriched in responders and closely related to *Clostridium trichophyton’s* abundance (100).

These findings suggest that the diversity and specific composition of intestinal microorganisms and changes in particular metabolites can be practical biomarkers to predict the clinical response and survival benefit of HCC immunotherapy. Moreover, certain microorganisms or metabolites enriched in non-responders or responders may become potential targets to improve the efficacy of immunotherapy for HCC. For example, combining acetate with a PD-1/PDL-1 blocker has significantly enhanced the anti-tumor effect (101).

In the intestines of healthy individuals, bacteria and fungi maintain homeostasis. Unfortunately, compared with bacteria, there is a lack of research on the role of commensal fungi in the ICIs immunotherapy of cancer. Studies that use bacteria to enhance the therapeutic efficacy of immune checkpoint inhibitors may disrupt the original bacterial-fungal homeostasis, and these studies have almost without exception ignored commensal fungi. Therefore, the symbiotic fungi group will be a very promising area in subsequent research.

### 3.2 Emerging viral-based immunotherapy

Oncolytic viruses (OVs) are an emerging class of cancer therapeutics with several advantages in cancer treatment (**Figure 1C**). Selectively infecting and destroying cancer cells while sparing normal cells, OVs result in minimal damage to healthy tissues (102, 103). This targeted approach stimulates an antitumor immune response by presenting tumor-associated antigens to the immune system (104). Additionally, OVs induce the expression of inflammatory and immunomodulatory cytokines in the tumor microenvironment, further enhancing the immune response (105). Efficacious against various malignant neoplasms, including drug-resistant lymphoproliferative diseases (106), OVs have shown promise in clinical trials with well-tolerated outcomes for cancer patients (107). Furthermore, OVs offer the potential for delivering multiple eukaryotic transgene payloads, inducing immunogenic cell death, and promoting antitumor immunity (108). Overall, this evolving approach in cancer therapy represents a promising avenue to provide added patients benefits.

Oncolytic viruses (OVs) have been extensively studied as promising cancer therapeutics. They were found to inhibit HCC growth, including directly dissolving tumor cells, exposing neoantigens, sequentially activating anti-tumor immunity (109). The most studied OVs are a vaccinia virus, JX-594 (110, 111). This engineered OVs, JX-594, have two genes inserted at TK gene region. One gene encodes human granulocyte-macrophage colony-stimulating factor (hGM-CSF) and the other gene encodes lac-Z (112, 113) to form this functional vaccinia virus. JX-594 can induce anti-tumor immunity and inhibit tumor blood vessels by promoting the maturation of myeloid cells and dendritic cells. Additionally, other appealing oncolytic vectors have been developed for the treatment of HCC, such as a new chimeric vector called recombinant VSV-NDV (rVSV-NDV) (114), and Ld0-GFP, based on herpes simplex virus type 1 (HSV-1) (115). Both vectors have shown excellent cancer cell killing ability and significantly improved the survival in the HCC mouse model from both *in vivo* and *in vitro* studies. Notably, the combination of oncolytic viruses with other immunotherapies like immune checkpoint blockade (ICB) and chimeric antigen receptors (CAR) has shown promising tumor treatment efficacy (116). Hence, the approach of combining the oncolytic viruses with ICB or CAR will bring new hope to HCC treatment.

Endogenous retroviruses (ERVs) are retroviral sequences that have become integrated into the genome of a host species. They are remnants of ancient retroviral infections that have been passed down through generations (117). ERVs can be found in the genomes of various organisms, including humans (118). The host has co-opted ERVs over time, and some ERV genes have been repurposed for host functions (119). ERVs are regulated by various mechanisms, including epigenetic modulation, and their dysregulation has been associated with neurological diseases, cancer, and inflammatory processes (120).

Endogenous retroviruses (ERVs) have several advantages in cancer immunotherapy (**Figure 1C**). First, ERV reactivation can induce an interferon response, known as viral mimicry, which sensitizes tumor cells to immunological recognition (121, 122). This viral mimicry state can be triggered by drugs or cellular changes in tumor cells (123). Second, ERV expression can lead to the production of tumor-specific antigens (TSAs). These TSAs, derived from translated Human ERV elements, contribute to the landscape of antigens recognized by the immune system (124, 125). They have been recognized by cytotoxic CD8+ T cells, resulting in cancer cell recognition. Third, the combination of viral mimicry and T-cell recognition can enhance the effectiveness of existing immune stimulatory therapies, such as checkpoint inhibition. In summary, the reactivation of ERVs in cancer cells has the potential to sensitize tumors to immunotherapy and improve treatment outcomes.

Recent advances have been made in the use of ERVs for cancer immunotherapy. Human ERVs represent 8% of the human genome and have been identified as potential tumor antigens for immunotherapy. Bioinformatic approaches have been developed to identify shared CD8+ T cell epitopes derived from cancer-associated HERVs in solid tumors. *In vitro* priming assays have confirmed the immunogenicity of these epitopes, leading to the induction of high-avidity CD8+ T cell clones. HERV-specific T cells, which have been shown to specifically recognize and kill tumor cells presenting HERV epitopes on HLA molecules, have also been identified among tumor-infiltrating lymphocytes from patients with breast cancer (126). Recent studies have shown that anti-ERV antibodies play a role in the effectiveness of ICB immunotherapy in mouse and human lung cancer, and nearly half of the LUAD patient samples showed antibody activity against the human endogenous retrovirus HERV-K. Among the 7 LUAD patients who received ICB, all had increased HERV-K envelope-reactive antibodies. Additionally, it was found that this increase in antibodies was positively correlated with survival after discontinuation of ICB (127).

## 4 The mechanistic studies of carcinogenic microbiome

**Figure 2:**
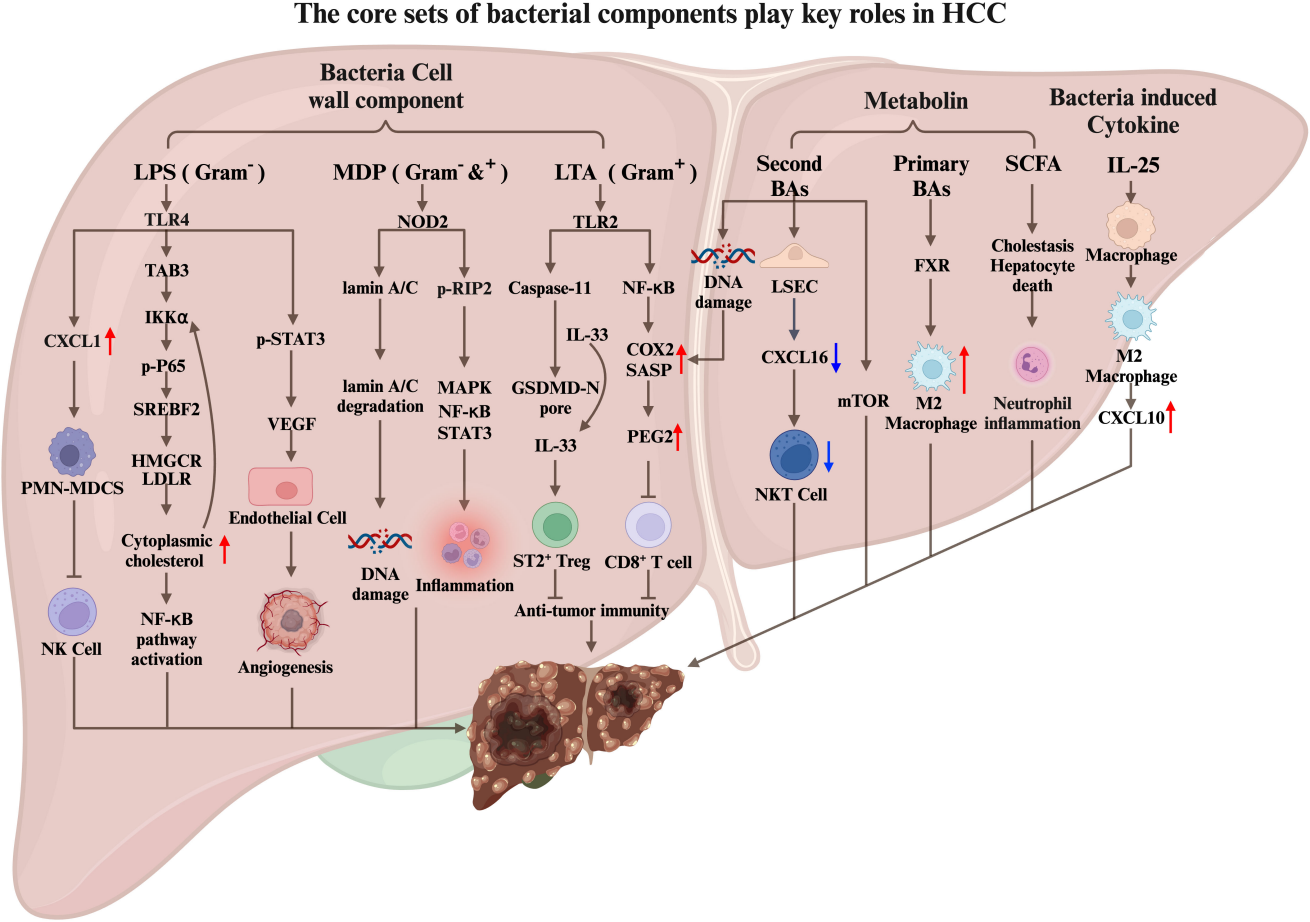
The core sets of bacterial components play key roles in HCC carcinogenesis. Bacterial cell wall components (LPS, MDP, LTA), bacterial-related metabolin, and bacterial-induced cytokines translocated to the liver through the portal vein regulate the liver cancer immune microenvironment by interacting with liver cells, tumor cells, and immune cells, thereby promote HCC tumorigenesis.

The relationship between intestinal microbiome and liver disease is reciprocal, in which the changes can contribute to liver disease (128). For instance, dysbiosis of gut microbiome and gut leakage can cause bacterial products to reach the liver through the portal vein, causing an inflammatory response and promote liver disease. Conversely, cirrhosis and portal hypertension can alter the composition of the intestinal microbiome, leading to the increased translocation to liver. Once translocated to liver, the microbiome can, in turn, alter the tissue immune microenvironment, resulting in more inflammation, which may finally lead to liver cancer. Notably, to advance our understanding by uncovering the underlying mechanisms and to identify potential therapeutic targets is imperative (**Figure 2**).

### 4.1 The core sets of bacterial components play vital roles in HCC carcinogenesis

The innate immune system plays a vital role in the host’s resistance to pathogenic bacterial infections as the second line of defense. Innate immune cells recognize Pathogenic Molecule-related Patterns (PAMPS) on the surface of pathogenic bacteria through pattern recognition receptors. This theory, proposed by Janeway in 1989 (129), is based on the idea that pathogenic molecule-related patterns on the cell wall of pathogenic bacteria, such as lipopolysaccharide, muramyl dipeptide, lipoteichoic acid, and peptidoglycan, or intracellular nucleic acid molecules. And the pattern recognition receptors are the receptors present on the innate immune cells, such as Toll-like receptors (TLR) and NOD-like receptors (NLR), which can recognize exogenous or endogenous dangerous molecules. Once relevant danger signals are recognized, a series of inflammatory responses are initiated, leading to the production of inflammatory cytokines and mediation of immune cell phagocytosis to eliminate corresponding pathogenic bacteria or abnormal cells. The above important findings lead to an intriguing note that the phagocytosis, which can be exploited as a cargo for the pathogenic bacteria to evade immune surveillance.

Intracellular bacteria are bacteria that live inside the cells of their host organisms. They can be found in various types of hosts, including corals, protists, arthropods, marine invertebrates, mammals, immune cells and cancer cells. These bacteria have developed many strategies to survive and travel within the host cells (130, 131). The presence of intracellular bacteria can be found in immune cells and other cell types in tumor (132). In addition, intracellular bacteria have been found to promote tumor metastasis (133).

Intracellular bacteria have developed multiple mechanisms to evade the host immune system (134). Moreover, bacteria can interfere signaling of immune cells to suppress the host’s immune response. Mycobacterium tuberculosis evades the immune response by inhibiting the NF-κB signaling pathway through the secretion of Rv0222 after being engulfed in immune cells (135). Taken together the interaction of intracellular bacteria with the host immune system can impact tumor progression.

#### 4.1.1 Lipopolysaccharide (Gram-negative bacteria)

LPS is a potent antigen and a Gram-negative bacteria cell wall component that can activate the host’s immune response, including sepsis. Additionally, LPS has been shown to promote tumor proliferation, angiogenesis, tumor invasion, and metastasis (136, 137). In mice, LPS stimulation significantly increases the expression of inflammatory genes and promotes the development of HCC (45). Moreover, high levels of circulating LPS in patients with chronic liver disease predispose them to HCC (138).

In the context of primary sclerosing cholangitis (PSC) and colitis-promoted cholangiocarcinoma (CCA) mouse models (139), LPS becomes a crucial promoting factor. Colitis induced by PSC and dextran sulfate sodium (DSS) disrupts intestinal homeostasis, leading to changes in intestinal permeability. Consequently, LPS, a bacterial cell wall component, enters the liver through the portal vein and binds to TLR4 receptors on hepatocytes. This binding stimulates liver cells to secrete CXCL1, which recruits CXCR2^+^ PMN-MDSCs and facilitates CCA progression. Inhibition of PMN-MDSCs impairs the tumor cell-killing function of NK cells, resulting in uncontrolled tumor growth. Conversely, inhibiting the LPS-TLR4 and CXCL1-CXCR2 pathways reduces PMN-MDSCs in liver tissue and significantly decreases tumor size. These findings highlight the role of intestinal imbalance, LPS production by gut microbiome, and PMN-MDSC recruitment in CCA development. Another study on CCA immunotherapy focused on improving anti-PD-L1 treatment efficacy against CCA using an orthotopic mouse CCA tumor model (140). CCA patients and orthotopic CCA mouse models respond poorly to immune checkpoint blockade. However, the simultaneous depletion of PMN-MDSCs and macrophages made CCA more responsive to anti-PDL1 treatment. These findings suggest that PMN-MDSCs could be potential targets for CCA immunotherapy. Further research is required to investigate whether regulating bacteria-induced PMN-MDSC production and reducing LPS components can enhance the effectiveness of anti-PD-L1 immunotherapy.

Tumor angiogenesis plays a crucial role in tumor proliferation and metastasis. HCC is a hypervascular tumor characterized by rich and tortuous blood vessels (141). Another way for LPS to promote the development of hepatocellular carcinoma is to promote tumor angiogenesis (136). LPS promotes HCC development by activating STAT3 in liver cancer cells through TLR4 receptors. This activation leads to increased expression of VEGF, which acts on neighboring epithelial cells and promotes angiogenesis. Moreover, VEGF can bind to VEGFR on tumor cells, activating STAT3 and facilitating tumor cell proliferation and migration. The complex microenvironment of hepatocellular carcinoma (HCC) is characterized by chronic inflammation. Abnormal cholesterol metabolism has been implicated in the physiology of liver cancer cells. *In vitro* co-incubation of LPS with liver cancer cells results in cholesterol accumulation (142). Mechanistically, LPS upregulates the expression of HMGCR, LDLR, and SREBF2 through the LPS/TLR4/TAB3/IKKα/NF-κB pathway. Cholesterol accumulation in cells can also enhance LPS/NF-κB-mediated inflammatory responses, potentially affecting the immune microenvironment of liver cancer.

#### 4.1.2 Muramyl dipeptide (both Gram-positive and Gram-negative bacteria)

MDP is an essential molecular pattern of pathogenic microbiome in both Gram-positive and Gram-negative bacteria. NOD2, known for recognizing MDP and activating inflammatory responses, plays a crucial role in this process (143). MDP was injected intraperitoneally in the mouse liver cancer model induced by DEN/CCL4. The results showed that MDP significantly increased the tumor incidence, burden, and size compared to the control group. These effects were found to be dependent on the NOD2 receptor. Mechanistically, MDP promotes the development of liver cancer through two pathways. The first pathway involves the activation of RIP2 downstream of NOD2, which activates the MAPK, NF-κB, and STAT3 signaling pathways, leading to increased liver inflammation. The second pathway is independent of RIP2 and involves the activation of the nucleus of cells by NOD2. This activation triggers autophagy, which promotes the degradation of lamin A/C. Reductions in lamin A/C impair the hepatocytes’ ability to repair damaged DNA, leading to increased genomic instability and tumorigenesis.

#### 4.1.3 Lipoteichoic acid (Gram-positive bacteria)

In a recent study, Naoko Ohtani and colleagues explored the role of lipoteichoic acid (LTA), a Gram-positive bacteria cell wall component, in developing liver cancer in mice fed a high-fat diet. They found that LTA, which can be translocated and accumulated in liver tissue, synergizes with the gut microbial metabolite deoxycholic acid to enhance the senescence-associated secretory phenotype (SASP) of hepatic stellate cells (HSCs). Toll-like receptor 2 (TLR2) plays a crucial role by upregulating the expression of SASP factors and COX2. COX2-mediated prostaglandin E2 (PGE2) production suppresses anti-tumor immunity through the PTGER4 receptor, promoting HCC progression (144).

Furthermore, the team discovered a novel pathway through which LTA promotes HCC (145). In the mouse model of obesity-induced HCC, aged hepatic stellate cells produce a significant amount of SASP, including IL-1β and IL-1β-dependent IL-33. The secretion of active IL-33 and IL-1β is mediated by LTA, which leaks from the intestine into the liver and is recognized by the TLR2 receptor on liver cells. This recognition activates caspase11, leading to the cleavage of gasdermin D (GSDMD) and the subsequent formation of GSDMD amino-terminal–mediated pores on the cell membrane. These pores promote the release of IL-33 and IL-1β. IL-33, once released by HSCs, activates ST2-positive Treg cells in the liver tumor microenvironment, thereby facilitating the development of HCC. Based on these findings, targeting the interaction between PGE2 and the PTGER4 receptor and inhibiting the formation of GSDMD-mediated pores could be potential therapeutic strategies for HCC.

#### 4.1.4 Bacterial metabolism

A significant amount of metabolites that act as crucial signaling factors and energy substrates are mutually produced by the host and the microbiome. Bile acids like deoxycholic acid (DCA) and lithocholic acid (LCA) are included in these metabolites, playing critical roles in digestion and mediating both health-promoting and disease-causing processes (146, 147). Also included are Short-chain fatty acids (SCFA) such as acetate and butyrate, which influence the intestinal microbiome’s composition, impact colonic function, and provide an energy source for both host cells and intestinal microorganisms (148, 149). The interaction between the host and this fluctuating group of tiny molecule metabolites, otherwise known as the metabolome, can influence the immune system, as well as different metabolic phenotypes, and could even affect factors of disease risk and responses to treatment (150, 151).

Liver cells produce bile acids that can be secreted into the intestine to aid lipid digestion, and bacteria in the intestine metabolize bile acids into secondary bile acids. Dysbiosis can change normal intestinal microbial metabolism, leading to the occurrence and development of liver cancer and the hepatic translocation of bacterial cell wall components. In a high-fat diet mouse model, the liver is susceptible to deoxycholic acid, causing DNA damage to liver stellate cells and promoting their aging. Liver cells produce bile acids that can be secreted into the intestine to aid lipid digestion. Bacteria in the intestine metabolize bile acids into secondary bile acids. An imbalance of the intestinal flora destroys the balance between primary and secondary bile acids. Antibiotics (ABX) can clear the intestinal microbiome, and in various mouse liver cancer models, mice treated with ABX had fewer and smaller tumors than the control group. Furthermore, ABX’s elimination of microbiome in tumor-bearing mice reduces the accumulation of secondary bile acids in the liver, increasing the amount of primary bile acids. The primary bile acids stimulate liver sinusoidal endothelial cells (LSEC) to secrete the chemokine CXCL16, recruiting CXCR6^+^ NKT cells to the liver to kill tumor cells. Additionally, a specific bacterium, *C.scindens*, was found to reduce the number of NKT cells in the liver, leading to an increase in liver tumors (152).

In a high-fat diet mouse model, antibiotic treatment also alleviates liver disease and inhibits tumor development while reducing secondary bile acids (153). The activation of the mTOR signaling pathway by secondary bile acids is associated with cancer, but further research is needed to determine its role in the occurrence of HCC (154).

The accumulation of primary bile acid TCA in the liver creates an immunosuppressive tumor microenvironment controlled by the metabolic regulatory gene Sirt5. Low expression of Sirt5 is associated with a poor overall survival rate of HCC patients. Knockdown of sirt5 increases the accumulation of TCA, promoting M2 macrophage polarization and the development of an immunosuppressive tumor microenvironment. Bile acid sequestrants can reverse the effects of Sirt5 deficiency in promoting M2-like polarized TAMs and liver tumor growth (155).

SCFA are produced by bacteria metabolizing dietary soluble fiber and are considered beneficial to health. However, diets rich in inulin, converted to butyrate by bacteria, may induce icteric HCC by causing cholestasis, hepatocyte death, and neutrophil inflammatory response. This process depends on intestinal microbiome. Using cholestyramine to block bile acids can inhibit the promotion of liver cancer by short-chain fatty acids (156).

#### 4.1.5 Bacterial microbes induced cytokines and chemokines

IL-25 levels are significantly elevated in HCC patients and are negatively correlated with survival rate after liver resection (157). Experimental mouse models have shown that IL-25 in the liver originates from epithelial tufted cells in the intestine. Dysbiosis of the intestinal flora, induced by vancomycin stimulation, leads to the proliferation of colon epithelial tufted cells, along with an increase in the expression of IL-25. Subsequently, IL-25 enters the liver through the portal vein and activates M2 macrophages, contributing to an immunosuppressive microenvironment within the liver. Additionally, the activated M2 macrophage and the secretion of the chemokine CXCL10 facilitate the occurrence, migration, and invasion of HCC.

### 4.2 Bacterial microbes in other types of carcinogenesis

Bacteria have been found to contribute to the advanced stages of HCC. Moreover, the role of bacteria in immune checkpoint blockade (ICB) is increasingly recognized. Hence, it is of utmost importance to examine the correlation between specific bacteria and the evolution of cancer. In light of this, we provide an overview of groundbreaking studies that probe the linkage between particular bacteria and cancer and further delve into the consequences of bacteria in the domain of ICB treatment. These reports serve as valuable references for future HCC translational research (**Figure 1A**).

#### 4.2.1 Bacteria in gastric cancer

Approximately half of the global population’s stomachs are colonized by a gram-negative, spiral-shaped bacterium known as *Helicobacter pylori* (158). In 1982, *Helicobacter pylori* has been discovered as the cause of gastric and duodenal ulcers (159). additionally, its presence in the stomach elevates the risk of gastric adenocarcinoma and peptic ulcer disease (160), making it the most potent known risk factor for gastric cancer, the world’s third highest cause of cancer-related fatality (161). An essential element in the carcinogenesis process is the cytotoxin-associated gene A (CagA) protein produced by *Helicobacter pylori*. The bacterial type IV secretion system transports this protein into gastric epithelial cells. Once delivered, CagA acts as an extrinsic scaffold or hub protein, disrupting multiple host signaling pathways that induce mammalian malignancies (162). Some of the specific mechanisms involve the stimulation of cell proliferation through mitotic signaling pathways, such as PI3K-AKT, SHP2, MAPK, and β-catenin-Wnt pathways (163–169). In addition, CagA stimulates the NF-kB signaling pathway (170, 171), which aids in attracting inflammatory cells, triggers damage through reactive oxygen species, and contributes to wound-healing responses. These effects collectively contribute to the development of cancer.

Current research has pinpointed nine gene mutations that elevate the likelihood of developing gastric cancer. The observations indicate that a *Helicobacter pylori* infection can amplify this cancer risk linked with these mutations. Consequently, it is of utmost importance to detect and eliminate Helicobacter pylori infections in individuals who carry these gene mutations related to an increased susceptibility to gastric cancer (172). Similarly, a study analyzing 1,043 *Helicobacter pylori* genomes found that the 171S-171L mutation of the serine protease HtrA is significantly associated with gastric cancer. This mutation increases the activity of proteolytic enzymes and the splitting of proteins in epithelial junctions, results in severe damage to the epithelial tissues, and aids in the delivery of the CagA oncoprotein into the epithelial cells. These events result in NF-kB-mediated inflammation, increased cell proliferation, and host DNA double-strand breaks, collectively triggering gastric carcinogenesis (173).

Additionally, research has also explored the involvement of other microorganisms in the development of gastric cancer, including *Lactobacillus*, *Lachnospiraceae*, and *Nitrospira*, among others (174–176).

#### 4.2.2 Bacteria in oral squamous cell carcinoma

Plaque accumulation in the oral cavity leads to the secretion of toxins by bacteria, causing gum inflammation and potentially progressing into periodontitis. *Porphyromonas gingivalis*, *Tannerella forsythia*, and *Fusobacterium nucleatum* are the primary bacteria involved in this process. These bacteria have also been linked to oral squamous cell carcinoma (177), with *Porphyromonas gingivalis* showing the strongest correlation (178). *Porphyromonas gingivalis* notably encourages cell growth, inhibits cellular self-destruction, stimulates the formation of new blood vessels, and aids in the spread and propagation of cancer cells, thereby playing a crucial role in the progression of oral squamous cell carcinoma (178, 179). Furthermore, the presence of *Porphyromonas gingivalis* in the oral cavity is also linked to a heightened risk of developing pancreatic cancer (180).

#### 4.2.3 Bacteria in colorectal cancer

Colorectal cancer (CRC), a prevalent digestive system malignancy, is noted for its high incidence and fatality rates. Its emergence and progression are intimately linked to the intestinal microbiome, in particular, the bacterial group, given the vast diversity of microbiomes like bacteria, fungi, and viruses that inhabit the gut. Bacteria such as *Fusobacterium nucleatum*, *Bacteroides fragilis*, and *pks^+^E. coli* found in the intestine are involved in promoting CRC, while others like *Streptococcus thermophilus*, *Streptococcus salivarius*, *Clostridium butyricum*, *Lactobacillus gallinarum*, and *Lactobacillus paracasei* are considered to inhibit CRC (181–183).

*Fusobacterium nucleatum*, a Gram-negative anaerobic bacterium, is prevalent in the human mouth (184), but appears less frequently in a healthy colorectal region (182). In colorectal patients, the abundance of *Fusobacterium nucleatum* is significantly increased (49, 185, 186). Research indicates that *Fusobacterium nucleatum*, which is associated with CRC, is transferred from the oral cavity to tumor tissues through the gastrointestinal tract (182, 187). Additional studies also demonstrate the crucial role of Fap2, a protein expressed by *Fusobacterium nucleatum*, in its localization to colorectal cancer tumor tissues (188). Questions arise about the entry pathways and liver colonization, particularly in tumor and immune cells, as commensal microorganisms may migrate and penetrate the liver via the portal vein during HCC development.

Mechanistically, *Fusobacterium nucleatum* promotes the occurrence and development of CRC through various pathways. It regulates CRC glucose metabolism by activating lncRNA ENO1-IT1 transcription, enhancing the glucose metabolism of tumor cells, and exerting a cancer-promoting effect (189).

The FadA adhesin in *Fusobacterium nucleatum* triggers the Wnt/β-catenin signaling pathway, which results in β-catenin entering the nucleus. This then enhances the expression of cancer-causing genes c-Myc and Cyclin D1, subsequently promoting the growth of tumor cells. Additionally, FadA upregulates annexin A1, which further enhances the effect of FadA on activating the Wnt/β-catenin signaling pathway. The combination of FadA and E-cadherin affects the immune microenvironment of CRC by promoting the expression of inflammatory genes (190, 191).

The latest research has identified ALPK1 as a pattern recognition receptor that recognizes *Fusobacterium nucleatum* and upregulates the expression of ICAM1 through the ALPK1/NF-κB signaling pathway, thus promoting the adhesion, extravasation, and metastasis of tumor cells (192).

Furthermore, *Fusobacterium nucleatum* is significantly associated with non-response to anti-PD-1 therapy in CRC patients. Higher abundance of *Fusobacterium nucleatum* and higher succinic acid content were observed in patients with metastatic colorectal cancer who did not respond to immunotherapy. Mechanistically, *Fusobacterium nucleatum*-derived succinic acid inhibits the cGAS-interferon-b pathway, limiting the trafficking of CD8^+^ T cells to the tumor microenvironment (TME) *in vivo* and inhibiting anti-tumor responses. However, the antibiotic metronidazole has been found to reduce the content of *Fusobacterium nucleatum* in the patient’s intestines, enhancing the re-sensitivity of tumors to *in vivo* immunotherapy (193).

#### 4.2.4 Bacteria in lung cancer

As the technologies of next-generation sequencing evolve, the microbiome analysis is becoming more routine. Research on the correlation between commensal microorganisms and lung cancer has focused on the diverse microbiome in the human lower respiratory tract (194). Certain research has examined the disparities in gut bacteria between non-small cell lung cancer (NSCLC) patients and healthy people. These studies have found that patients with NSCLC exhibit dysbiosis in their intestinal flora, with significant upregulation of *Prevotella*, *Gemmiger*, and *Roseburia* at the genus level (195). Airway brushing samples were gathered in advance from patients undergoing bronchoscopy in a separate study. Among the samples, 39 were confirmed to have lung cancer, 36 did not have lung cancer, and 10 airway brushings from a healthy control group were included. A detailed examination of the microbial makeup in these samples unveiled a substantial presence of *Streptococcus* and *Veillonella* in the lower respiratory system of patients with lung cancer. Additionally, these bacteria were found to be associated with the upregulation of ERK and PI3K signaling pathways (196). Subsequent studies further discovered that *Veillonella* is linked to the upregulation of IL17, PI3K, MAPK, and ERK pathways (197). Furthermore, *Streptococcus* and *Veillonella* species were significantly enriched in the intestines of patients with pancreatic cancer (198).

A Japanese research team also conducted clinical research on applying probiotics in treating NSCLC. They found that a significant extension in progression-free survival (PFS) and overall survival (OS) rates could be achieved in advanced NSCLC patients receiving ICB treatment by utilizing *Clostridium butyricum MIYAIRI 588* (199). In the Lewis lung cancer mouse model, intragastric administration of *Akkermansia muciniphila* was observed to inhibit tumor occurrence significantly. Metabolome analysis revealed that the possible mechanism behind this inhibition is the reprogramming of tumor metabolism by *Akkermansia muciniphila* (200).

### 4.3 Emerging mechanistic studies highlight the role of fungi in the following tumor types

Fungi, such as Candida, Saccharomyces, and Malassezia, are essential components of human commensal microbiome, colonizing various niches within the human body. The gut and other mucosal surfaces are typical habitats for these fungi (17, 201, 202). *Candida* species predominantly colonize the oral cavity, while *Malassezia* dominates the skin (203, 204). Despite accounting for only 1% of the human commensal microbiome, fungi significantly impact the host’s health and disease (17). They can act as opportunistic pathogens and infect immunocompromised patients, including those with cancer (24). Notably, their effects should not be overlooked (**Figure 1B**). The host’s natural immune system identifies fungi through their microbe-related molecular patterns, notably the carbohydrate components of the fungal cell wall. The detection of these patterns are carried out by pattern recognition receptors, such as C-type lectin receptors, found on epithelial and myeloid cells. Receptors such as Dectin, Lectin, Mincle, Toll-like receptors, and Nod-like receptors (NLR) activate signaling mechanisms involving SYK/CARD9, SYK/PLCy2, MYD88, and TRIF when they bind (205). Consequently, this triggers the production of signaling molecules, including interleukin 1β(IL-1β), IL-6, IL-12, IL-23, TGF-β, and interferon γ, which stimulate the immune response of T helper 1(Th1) and Th17 cells (206).

#### 4.3.1 Fungi in HCC progression

There has been less research on the mechanism of fungi in the occurrence and development of liver cancer than bacteria. However, as early as the last century, it was proven that aflatoxin, a toxin produced by fungi of the *Aspergillus* genus, can cause liver cancer. In simple terms, aflatoxin promotes the mutation of codon 249 of the P53 gene (207). Recent studies have also identified AhR as the critical factor that mediates the cytotoxicity of aflatoxin (208). Additionally, *Candida albicans* and *Malassezia* have been reported to regulate the progression of HCC (41, 65). Mechanistically, abnormal colonization of *Candida albicans* reprograms the metabolism of HCC and promotes its advancement in an NLRP6-dependent manner.

Fungi’s role in HCC is still poorly understood. However, the study of the relationship between fungi and cancer is inevitable. Therefore, we pay more attention to the underlying mechanisms between fungi and different cancer types, and try to get the inspirations from these mechanisms, which can be applicable in the HCC field.

#### 4.3.2 Fungi in oral cancer

*Candida* species are normal commensals of the oral cavity in almost 100% of the healthy population (209). Candidiasis, the most frequent opportunistic fungal infection, is caused by *Candida albicans*. This fungal species can catalyze the oxidation of ethanol into acetaldehyde, a class of carcinogens. Moreover, *Candida albicans* isolated from oral cancer patients exhibit a more vital ability to catalyze acetaldehyde production (210). Another carcinogenic metabolite produced by *C. albicans* is N-nitrosobenzylmethylamine. In an oral cancer model, supplementation of rats with a nitrosamine-producing strain of *C. albicans* resulted in a robust carcinogenic effect (211). *Candida albicans* is a dimorphic fungus that can exist in two forms: yeast or hyphae (212). The tip of the hyphae releases a cytolytic peptide toxin called Candidalysin, which activates the mitogen-activated protein kinases (MAPK) pathway and damages the oral epithelium. This process triggers the release of pro-inflammatory cytokines and is believed to play a role in tumorigenesis (213, 214).

#### 4.3.3 Fungi and colorectal cancer

CARD9 is a signaling adaptor protein involved in the transduction of signals from various innate pattern recognition receptors, including the C-type lectin receptors, intracellular NOD receptors, and nucleic acid sensors. It is also a critical molecule in the host immune response against fungal infections (215). In 2018, two studies reported that CARD9-deficient mice had increased tumor burden in the AOM/DSS-induced CRC mouse model (48, 55), but the underlying mechanisms differed. Malik et al. reported a fungal signaling cascade in myeloid cells mediated through SYK and CARD9 signaling that drives inflammasome activation and IL-18 maturation, promoting epithelial barrier repair, activating intestinal CD8^+^ T cells, and producing IFN-γ. This study found that myeloid cell-specific deletion of CARD9 or SYK reduces inflammasome activation and interleukin (IL)-18 maturation, increasing susceptibility to colitis and colorectal cancer in wild-type mice. In a model of enteritis induced by AOM-DSS, more severe colitis and more tumors were also observed using amphotericin B or itraconazole to eliminate commensal fungi, indicating that commensal fungi exert a protective effect.

On the other hand, Wang et al. found that the mycobiome of CARD9^−/−^ mice was altered and expanded and showed that *C. tropicalis* was the predominant fungus responsible for the increased tumor burden in this model. *Candida tropicalis* proliferates in CARD9-deficient mice, thereby inducing the differentiation and activation of MDSC cells, mainly G-MDSC, which is conducive to tumor immune evasion. Treatment with Ly6G antibodies or the anti-fungal drug fluconazole can significantly improve tumor incidence in CARD9-deficient mice. In follow-up studies, Wang et al. found that Dectin-3-deficient mice resulted in tumorigenesis and increased *Candida albicans* load after chemical induction. The proposed mechanism involves *Candida albicans* promoting the enhancement of glycolysis in macrophages, which leads to increased IL-7 secretion, thereby triggering aryl hydrocarbon receptors and STAT3 to induce RORγt^+^ innate lymphoid cells ILC3 to produce IL-22, promoting tumor progression.

Interestingly, Dectin-3 also plays a vital role in *Candida tropicalis*, promoting colorectal carcinogenesis. *Candida tropicalis* can significantly enhance the glycolysis level of MDSC cells. Mechanistically, *C.tropicalis* enhances the interaction between Syk and PKM2 in MDSCs, which results in the phosphorylation of PKM2 at Tyr105. PKM2 Tyr105 phosphorylation is essential for PKM2 nuclear translocation. Subsequently, atomic PKM2 functions as a coactivator of HIF-1α to promote HIF-1α-dependent expression of glycolytic enzymes, such as GLUT1, HK2, PKM2, LDHA, and PDK1, which in turn promotes aerobic glycolysis and the expression of iNOS, COX2, and NOX2, as well as the secretion of nitric oxide (NO) and reactive oxygen species (ROS), promoting the immunosuppressive function of MDSCs and thereby promoting the occurrence of colorectal cancer. Blocking PKM2 nuclear translocation can weaken *Candida tropicalis*-mediated colorectal cancer (216). Notably, inhibition of PKM2 via gene silencing or the drug shikonin renders HCC and patient-derived cell lines (PDC) responsive to chemotherapy-sensitive (217). At the same time, Dectin-3 also mediates Candida tropicalis to activate the NLRP3 inflammasome in MDSCs (218). The activation of NLRP3 depends on the mitochondrial reactive oxygen species generated by enhanced glycolysis and the JAK/STAT1 signaling pathway, which plays a role in AOM/DSS CRC. Pharmacological inhibition of the NLRP3 inflammasome rescues *C. tropicalis*-induced increased tumor burden in mouse models.

#### 4.3.4 Fungi and pancreatic cancer

Aykut et al. demonstrated that in patients with pancreatic cancer, the content of intratumoral fungi was significantly increased. They also found that *Malassezia globosa* was enriched in the mouse pancreatic cancer model. The elimination of the fungal biome resulted in reduced tumor growth, while *Malassezia globosa* was found to be enriched in tumors. Additionally, recolonization by chromobacteria accelerated tumor growth. The promotion of tumorigenesis by fungi was mediated through the activation of the complement cascade by binding mannose-binding lectins to glycans in the fungal cell wall (219). The presence of fungi was confirmed in a mouse model of PDAC using 18S ribosomal RNA (rRNA) sequencing and fluorescence *in situ* hybridization (56). Fungi were found in the PDAC mouse model, *Malassezia*, and another fungus, *Alternaria*, was detected. Treating mice with the anti-fungal agent amphotericin B significantly reduced tumor burden, while administration of *M. globosa* or *A. Alternaria* promoted tumor growth. Mechanistically, the KRAS G12D mutation increased the expression of IL-33, a damage-related molecule known as a danger-associated molecular pattern (DAMP) protein. The secretion of IL-33 is tightly regulated by cells to avoid immune responses (220). Intratumoral fungi can induce the secretion of IL-33 from tumor cells. Once secreted, IL-33 interacts with the cognate receptor ST2 to recruit TH2 and ILC2 cells, producing cytokines such as IL-4, IL-5, IL-13, and others, promoting pancreatic cancer progression. Genetic deletion of IL-33 or anti-fungal treatment resulted in robust PDAC tumor regression. The potential mechanism by which fungi induce IL-33 secretion involves the Dectin-1/CARD9 pathway.

#### 4.3.5 Fungi and the progression of lung cancer

Abnormal colonization of the lungs by *Aspergillus* due to CARD9^S12N^ mutations causes allergic bronchopulmonary aspergillosis (221). Furthermore, *Aspergillus sydowii* has been detected in tumor tissues of patients with lung cancer (59). In human samples analysis, it has been found that abnormal colonization of *Aspergillus sydowii* is associated with poor patient prognosis. In mouse experiments, researchers observed abnormal colonization of *Aspergillus sydowii* in the tumor microenvironment and an accumulation of MDSC cells and PDL-1^+^ CD8^+^ T cells. This process is mediated by the β-glucan found on the surface of *Aspergillus sydowii*, which promotes the production of IL-1β by macrophages. The specific pathway involved is the β-glucan/Dectin-1/CARD9 pathway, and the accumulation of MDSC cells and PDL-1^+^ CD8^+^ T cells contributes to the progression of lung cancer.

### 4.4 The mechanistic studies of viral infections in HCC carcinogenesis

The Hepatitis B Virus (HBV) contributes to HCC through several processes. The HBV X protein (HBx) is instrumental in the propagation and spread of HCC (222). HBx interacts with a protein called the suppressor of cytokine signaling 1 (SOCS1), which inhibits the breakdown of p65, a component of NF-κB. This interaction activates factors associated with the epithelial-mesenchymal transition (EMT), a significant process in the migration and invasiveness of cancer cells (223). HBV infection also promotes disease progression through mechanisms such as HBV gene integration, genomic instability, activation of cancer-promoting signaling pathways, and alteration of hepatocellular physiology (224, 225). Additionally, miR-142-3p, highly expressed in HBV-infected HCC patients, promotes HBV-infected M1-type macrophage ferroptosis, affecting the production of reactive oxygen species and accelerating HCC development. The complexities of HBV-induced HCC mechanisms are illuminated by these findings, which also suggest possible therapeutic targets for intervention HCV infection induces HCC also through a variety of mechanisms. Chronic inflammation, oxidative stress, insulin resistance, endoplasmic reticulum stress, hepatic steatosis, and liver fibrosis are all included in this category (226). HCV-related proteins, such as HCV core, E1, E2, NS3, and NS5A, dysregulate cell cycle and metabolism by modulating signal pathways (227). HCV infection also leads to genetic and epigenetic modifications, including host genetic factors, dysregulation of signaling pathways, and histone and DNA modifications (228, 229). In addition to causing genetic and epigenetic changes, such as alterations in host genetic elements, disruption in signaling pathways, and changes in histones and DNA, HCV infection also results in excessive production of reactive oxygen species (ROS) and diminished function of natural antioxidants. This may cause damage to the DNA, lipids, and proteins (230). ROS activates signaling cascades, and the activity of transcription factors are modulated, which leads to changes in gene expression associated with cell survival, proliferation, angiogenesis, invasion, and metastasis. These processes play a role in the advancement and evolution of HCV-related HCC.

## 5 Perspective and discussion

Liver cancer is one of the most lethal cancer types due to lack of effective early diagnosis. There are several steps, we may take to improve the survival of HCC. First urgent step is to improve the early diagnosis of HCC (3). Second is more effective classification of responders and non-responders are needed to HCC immunotherapy (90). In addition, tumor barrier obstructs the infiltration of immune cells (231). To address the above issues in HCC and microbiome may provide a key translational breakthrough, as a bulk of data from melanoma study supports this inference.

Current research on the relationship between cancer and microbiome has some limitations. Firstly, there needs to be standardized parameters to describe microbial composition, making it difficult to compare and harmonize different studies for meta-analysis (232). Secondly, many findings require validation in high-quality, preferably prospective, epidemiologic studies (233). Thirdly, the complexity and diversity of the microbiome make it challenging to establish causal relationships between specific microorganisms and cancer development (234). Additionally, the microbiome is highly individualized, and there is a need to consider inter-individual variation when studying its association with cancer (235). Furthermore, the microbiome is influenced by various factors such as diet, lifestyle, and environmental exposures, which can confound research findings (236). Lastly, there is a need for increased accuracy and reproducibility of data linking the microbiome to cancer.

Similarly, in preclinical research, the collection and processing of patient samples also face challenges. The most serious problem is “genomic contamination”, which will affect the results’ authenticity and the conclusions’ reliability. The latest report focuses on 2019 studies that have raised questions (219). Furthermore, the results of the re-analysis showed that there were very few sequencing reads belonging to fungi in pancreatic tissue, and there were no significant differences in the pancreatic or intestinal microbiome between healthy people and PDAC patients (237). At the same time, articles published in 2023 (238, 239) questioned the report published by Rob Knight’s team in Nature magazine in 2020 (22). The main questioning point is that it may be due to sequencing errors and low quality, genetic mutations, and so on, causing some human genome reads to fail to be successfully compared and thus mistakenly identified as microbial reads. Therefore, when processing and analyzing trace microbial samples, special attention should be paid to sample contamination issues, and multiple control groups should be set up to ensure the authenticity of the data.

In the process of combining clinical samples, it is also necessary to pay attention to the polymorphisms of genes related to the host’s immune response to pathogens (240), For example, CARD9 is a crucial adapter protein for host recognition and elimination of fungi. Changes in its function will make the host more susceptible to infection with fungi (241), In the *Aspergillus fumigatus* infection model, the CARD9^S12N^ mutation will be connected to CLR to activate the non-canonical NF-κB pathway, thereby producing a type 2 immune response (221), At the same time, CARD9^S12N^ is also related to the intestinal microbial composition of IBD patients, with a strong correlation, particularly in patients with homozygous mutations in CARD9^S12N^, where almost no Pichia is present in the sigmoid colon (242). It can be speculated that in cancer patients, gene mutations related to the immune response to pathogens seem to make patients more susceptible to related microorganisms, and microbial homeostasis will also be altered, leading to a worse prognosis.

High levels of variability both within tumors and between patients are distinguishing features of HCC (243). The intertumoral and intratumoral heterogeneity is notably seen in the differences in the genetic constitution (244, 245) and the distinct microenvironments within the tumor (246, 247). As previously mentioned, the commensal microbiome in HCC patients differs significantly from that of healthy individuals. Furthermore, various research groups have reported disparities in the composition of the commensal microbiome in HCC patients. Hence, the heterogeneity observed in HCC may also manifest in the diversity of commensal microbiome within patients. Past research has shown that if there is a disruption in the balance and permeability of the intestines, it may allow microorganisms or metabolites from the gut to penetrate the liver. Considering the anatomical and physiological connections between the liver and gastrointestinal tract, microorganisms and metabolites translocated to the liver activate the hepatic immune response, causing changes in the hepatic immune microenvironment. As a result, it could affect the development of HCC. Hence, along with traditional tumor markers like AFP and GPC3 (3), it is recommended that colonoscopy should be incorporated into the clinical assessment of HCC patients for the evaluation of the intestinal barrier’s integrity. Furthermore, when administering immunotherapy, it is crucial to monitor commensal microorganisms in patients. Patient saliva, tissue biopsies, blood, and stool samples should be collected before and after treatment. These samples should then be subjected to analyses, including metagenomics, viral profiling, and metabolite detection, to analyze the patient’s commensal microbial profile systematically. This analysis will help to establish a signature that can predict host tumor progression and enable accurate treatment planning based on the patient’s unique commensal microbiome profile. Precisely prediction is significantly important in administering ICB combined with the commensal microbiome or components. To enhance treatment effectiveness, characteristics, antifungals, antibiotics, bile acid sequestrants, probiotics, and prebiotics may be considered. Furthermore, prioritizing the maintenance of intestinal mucosa health is crucial.

**Figure 3:**
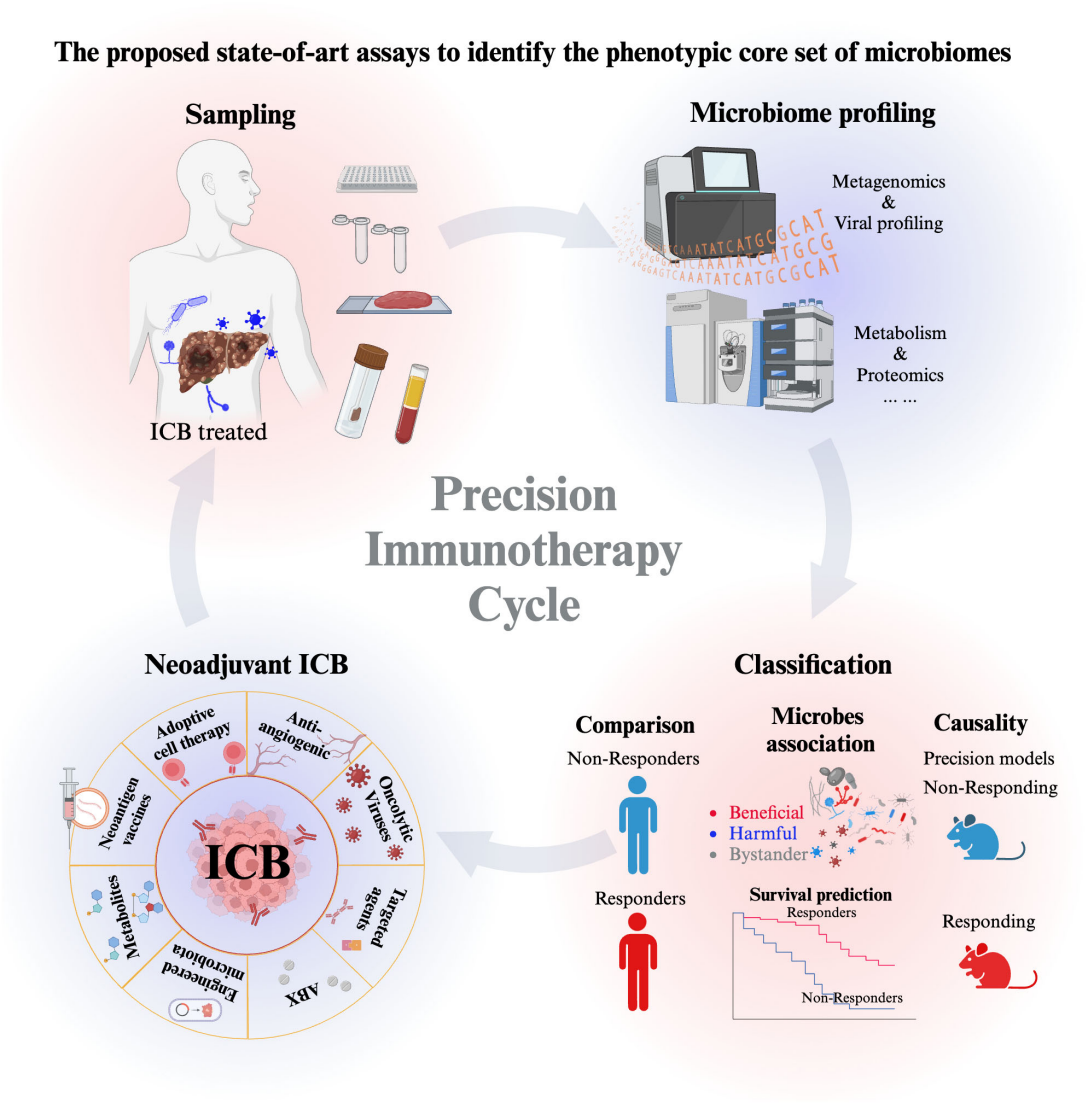
The proposed state-of-art assays to identify the phenotypic core set of microbiomes (bacteria, fungi, viruses) as robust biomarkers for HCC early diagnosis and treatment. The start of the precision immunotherapy starts from sampling, which includes saliva samples, blood samples, stool samples, and biopsy tissue samples. Then Microbiome profiling includes the high-throughput sequencing and the mass spectrometry to detect metabolism and proteomics. Further analysis will classify the microbe as beneficial, harmful and bystander microorganisms to indicating their association with the responder and non-responder patients. Moreover, the causality of beneficial and harmful microbe is further characterized in anti-tumor immunity in response to ICB. Based on the association and causal evidence, the optimized neoadjuvant strategies of ICB will be administrated to responders and non-responders. The precision immunotherapy cycle help to repeat the optimization in the cycling way from patients sampling to patient treatment. It continues to motivate and enlarge our deep understanding the microbe-host interplay, which ultimately optimizing the translational research of HCC immunotherapy.

To conclude, we propose to perform state-of-art assays to identify the phenotypic core set of microbiomes (bacteria, fungi, viruses) as robust biomarkers for HCC early diagnosis and treatment and to obtain a deep insight into the interaction mechanism between microbiome and intrinsic factors using precision mouse models (**Figure 3**). These understandings and precision immunotherapy cycling can be further translated into new forms of synthetic interventions for HCC immunotherapy.

## Author contributions

JL: Conceptualization, Funding acquisition, Supervision, Writing – original draft, Writing – review & editing. TL: Writing – original draft, Writing – review & editing. YG: Writing – review & editing. YL: Writing – review & editing.
